# Sustainable Agriculture by Increasing Nitrogen Fertilizer Efficiency Using Low-Resolution Camera Mounted on Unmanned Aerial Vehicles

**DOI:** 10.3390/ijerph16203893

**Published:** 2019-10-14

**Authors:** Dong-Wook Kim, Tae-Sun Min, Yoonha Kim, Renato Rodrigues Silva, Hae-Nam Hyun, Ju-Sung Kim, Kyung-Hwan Kim, Hak-Jin Kim, Yong Suk Chung

**Affiliations:** 1 Department of Biosystems & Biomaterials Science and Engineering, College of Agriculture and Life Sciences, Seoul National University, Seoul 08826, Korea; dwk8033@naver.com; 2 Department of Animal Biotechnology, Jeju National University, Jeju 63243, Korea; tsmin@jejunu.ac.kr; 3 Plant Bioscience, School of Applied Biosciences, Kyungpook National University, Daegu 41566, Korea; kyh1229@knu.ac.kr; 4 Institute of Mathematics and Statistics, Federal University of Goias, Goiania 74001-970, Brazil; renato.rrsilva@gmail.com; 5 Department of Plant Resources and Environment, Jeju National University, Jeju 63243, Korea; hnhyun@jejunu.ac.kr (H.-N.H.); aha2011@jejunu.ac.kr (J.-S.K.); 6 National Institute of Agricultural Sciences, Rural Development Administration (RDA), Jeonju 54874, Korea; biopiakim@korea.kr

**Keywords:** nitrogen fertilizer, chlorophyll contents, near infrared, normalized difference vegetation index (NDVI)

## Abstract

Nitrogen use efficiency in modern agriculture is very low. It means that a lot of synthetic chemicals are wasted rather than utilized by crops. This can cause more problems where the soil surface is thin and rocky like Jeju Island in the Republic of Korea. This is because overly used nitrogen fertilizer can be washed into the underground water and pollute it. Thus, it would be important to monitor the nitrogen deficiency of crops in the field to provide the right amount of nitrogen in a timely manner so that nitrogen waste can be limited. To achieve this, the normalized difference vegetation index (NDVI) was used to monitor chlorophyll content, which is tightly associated with nitrogen content in the buckwheat field. The NDVI was calculated with the data obtained by a low-resolution camera mounted on an unmanned aerial vehicle. The results showed that the NDVI can estimate the chlorophyll content of buckwheat. These simple but clear results imply that precision agriculture could be achieved even with a low-resolution camera in a cost-effective manner to reduce the pollution of underground water.

## 1. Introduction

Modern agriculture management depends on petroleum-based fertilizers especially for nitrogen supply. However, nitrogen use efficiency (NUE) in crop field is low [1]. There could be many reasons why there is low NUE with current nitrogen management practices. One of the major reasons is poor synchrony between soil nitrogen and crop demands due to uniform N applications to spatially variable soil nitrogen content [2]. In order not to fail nitrogen supply in the field due to the reasons above, higher input of nitrogen fertilizer tends to be applied in commercial practices, which causes unnecessary carbon-dioxide release and fossil fuel consumption. This is because most nitrogen fertilizers are based on petroleum, which could cause the potential adverse effect of global warming on ecosystems as disruptive urban land uses do [3,4]. Furthermore, the excess fertilizer is washed down to be drained and contaminates underground water, which could be a crucial issue, especially in the fragile water ecology of Jeju Island in the Republic of Korea, which resulted from Cenozoic volcanic activities [5]. This island is predominantly composed of highly porous and permeable volcanic rocks such as basalt, andesite, and trachyte. Such volcanic rocks are covered by relatively thin soil layers [6,7]. Consequently, there is little sustainable stream flow on the island because rainwater immediately infiltrates into the ground due to high permeability of the volcanic rocks, although it has heavy rainfall in summer [8]. This implies that a high level of buffer for applying chemicals including nitrogen fertilizer is required to conserve the environment on this island. Thus, an alternative strategy must be introduced, not only because of saving costs on fertilizers, but also because of managing environmental issues in this case. It is important to note that agriculture is already recognized as one of the reasons for global biodiversity loss, which makes low NUE a more important matter [9].

Most leaf chlorophyll contains nitrogen [10]. Thus, chlorophyll content can be a strong indicator for nitrogen content that could affect yield [11]. Among many kinds of methods to measure chlorophyll content, the normalized difference vegetation index (NDVI) is widely adapted using remote sensing technologies. This is achieved using unmanned aerial vehicles (UAVs) with multispectral sensors and cameras mounted on them. However, the price of the UAV itself as well as sensors and cameras to get high resolute data have been obstacles to do such studies. This is because UAV for agricultural study and sensors for high-resolution are thousands of US dollars.

Thus, this study is aimed to investigate if the images taken from low-resolution camera mounted on a low-cost UAV could estimate chlorophyll content in a crop planted in an open field condition. If this method is proved to be solid, nitrogen content in the leaf can be estimated by chlorophyll estimation using the NDVI. As a result, precision agriculture in an acceptable manner could be achieved. Even a few percent of improvement in NUE can be beneficial, not only to crop yield, but also to conserve the environment, especially delicate ecology such as Jeju Island.

## 2. Materials and Methods

### 2.1. Test Plots

**Figure 1:**
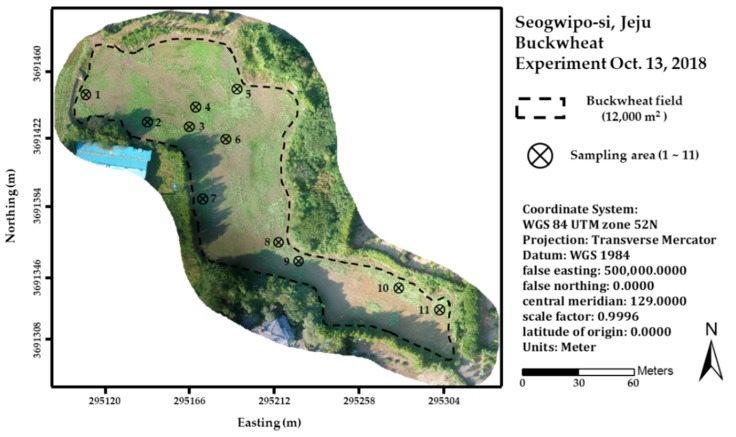
Test site: Buckwheat experiment.

A field experiment was conducted during the 2018 growing season in a buckwheat field (33.33° N, 126.80° E) located in Sehwa-ri, Seogwipo-si, Jeju, Republic of Korea (Figure 1). Seeds of buckwheat cultivar (120 kg·ha^−1^), Dasan, were scattered to be sown on 27 August 2018. The whole field size is around 12,000 m^2^. Before sowing, tillage was executed twice to prepare the field on 14 and 21 August 2018. Fertilizer (nitrogen:phosphorus:potassium = 21:17:17) of 300 kg·ha^−1^ was applied on 21 August 2018.

### 2.2. Unmanned aerial vehicles (UAVs) Flight and Image Acquisition

**Figure 2:**
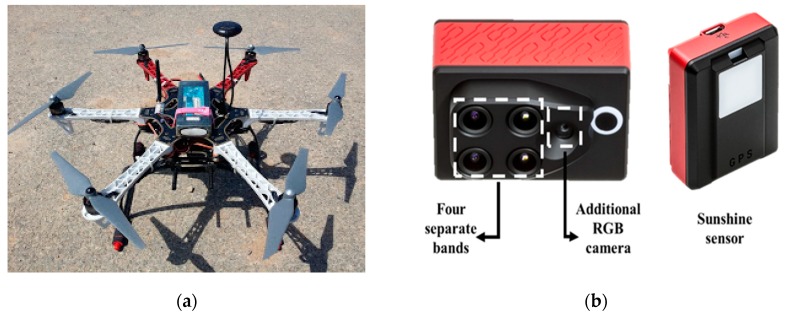
Views of (**a**) the manufactured unmanned aerial vehicle and (**b**) the commercial multi-spectral camera with sunshine sensor.

Images were taken on 14 October 2018 using a manufactured DJI hexa-rotor (Figure 2a, DJI F550, DJI, Shenzhen, Guangdong, China, material cost: around $1,100 USD). The platform had a total weight of 1.8 kg including Li-Po battery (14.8V 6000mAh 4S1P 35C, TATTU, Dublin, CA, USA) and an additional payload capability of 0.6 kg. The UAV was set to automatically fly over the experimental field using an automatic flight controller (Pixhawk, 3D Robotics, Berkeley, CA, USA), global positioning system (GPS), and inertial measurement unit (IMU). ArduPilot Mission Planner, an open source autopilot software for UAV, was used to generate and monitor autonomous flight paths in UAVs in real time. The flight path was designed to ensure an overlapping ratio of at least 85% for forward overlap and 70% for side overlap, which is necessary to identify sufficient key points for image orhomosaic. A 433 MHz wireless communication was used to monitor the UAV’s flight status and flight path, and a 2.4 GHz wireless remote controller was used for manual takeoff and landing.

A commercial multi-spectral camera (Sequoia, Parrot, Paris, France, material cost: around $3,500 USD) with sunshine sensor was used for image acquisition of buckwheat (Figure 2b). The multi-spectral camera was composed of four separate spectral sensors of Green (550 ± 20 nm), Red (660 ± 20 nm), Red Edge (735 ± 5 nm), and Near Infrared (790 ± 20 nm), with a definition of 1,280 by 960 pixels. An additional red, green, and blue (RGB) camera with a definition of 4608 by 3456 pixels was used to collect images and data of the test site. The signals obtained by the camera were corrected based on a UAV-mounted sunshine sensor for obtaining a precise image even under changing light conditions. The calibration process was built in the camera, so the corrected signal was stored as an image. The internal camera parameters, such as radial distortion, were auto-compensated by processing the bundle block adjustment in Pix4Dmapper (Pix4Dmapper Pro 3.0.17, Pix4D, Lausanne, Switzerland) [12].

Experimental buckwheat images were collected in the test site using the UAV platform with the multi-spectral camera and the sunshine sensor at 2 m·s^−1^ at 80 m above ground level (AGL). The details of the UAV flight experiment are shown in Table 1. The images were acquired based on a time-lapse function of the camera that took one image every one and half seconds to ensure an image overlapping ratio in the automatic flight mission.

### 2.3. Image Processing

**Figure 3:**
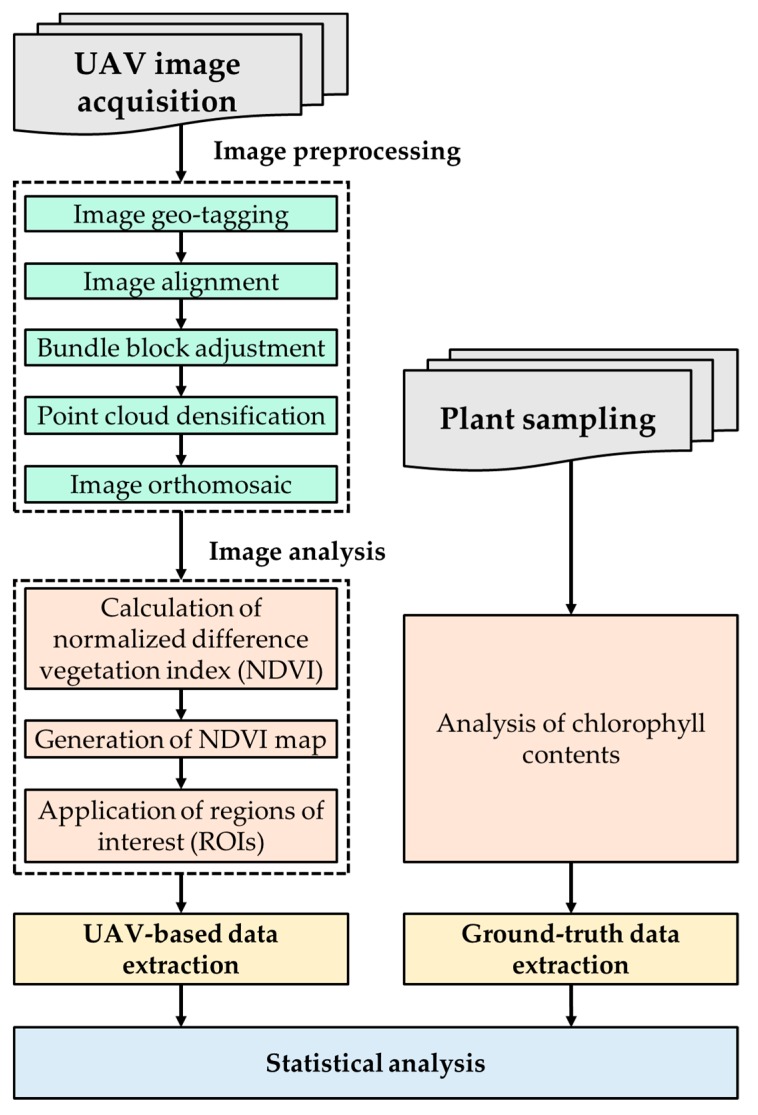
Flow chart of the image preprocessing and analysis steps.

Figure 3 shows the flow chart of the image preprocessing and analysis steps, including image orthomosaic, calculation of the NDVI, and statistical analysis. The Pix4Dmapper Pro 3.0.17 was used to process the collected multi-spectral and RGB images and corresponding orthomosaics. As such, an image geo-tagging process, that inserts the geographical information in the UAV-based images, is needed prior to initiating the software workflow for image orthomosaic. In the case of the used camera, GPS and IMU are built in, so flight position and attitude information is automatically recorded and geo-tagged to the collected images. After the geo-tagging process, the image processing workflow is composed of two main stages: (i) image alignment and feature points extraction; and (ii) orthomosaic generation.

**Figure 4:**
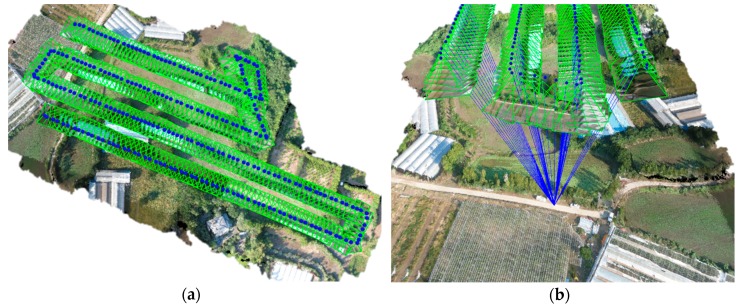
(**a**) Image alignment performed in this study, and (**b**) the extracted one 3D point from multi viewpoints of the aligned unmanned aerial vehicle (UAV) images.

As a first step, the geo-tagged images were aligned along the flight path (Figure 4a). As shown in Figure 4b, given a set of images depicting a number of 3D points from different viewpoints, bundle block adjustment can be defined as the problem of simultaneously refining the 3D coordinates describing the scene geometry and the parameters of the relative motion, and the optical characteristics of the camera employed to acquire the images, according to an optimality criterion involving the corresponding image projection of all points. The bundle block adjustment was used for feature-based 3D reconstruction algorithm in the image orthomosaic process. It amounts to an optimization problem on the 3D structure and viewing parameters, i.e., camera pose and radial distortion, to obtain a reconstruction which is optimal under certain assumptions regarding the noise pertaining to the observed image features. Therefore, the 3D position of point clouds extracted from multi-view 2D images can be calculated. After that, point cloud densification is necessary because unevenly distributed point clouds may lose detail and precision in the sparse areas. Finally, based on the extracted and matched feature points, the generated dense point cloud is interpolated to conduct an orthomosaic generation procedure with the image exported in GeoTiFF data format (*.tif).

After image orthomosaic, NDVI maps could be generated by calculation of the NDVI with the band math function in the ENVI software (ENVI 5.4, Harris Geospatial Solutions, Broomfield, CO, USA). To obtain ground truth data on the chlorophyll content of buckwheat, randomly selected crops from the test site were manually removed after the UAV flight. In order to effectively perform bivariate analysis between the UAV-based NDVI and ground truth data for buckwheat, 1 m circular regions of interest (ROIs) that represent the area of each grid were used.

### 2.4. Near Infrared (NIR) Vegetation Index

In this study, the NDVI based on the Near Infrared (NIR) channel was tested for estimating chlorophyll content of buckwheat in the mosaicked image. The NDVI is the most popular VI widely used for vegetation monitoring applications such as crop nutrient deficiency, long-term water stress, and evapotranspiration. The NDVI was calculated by following Equation (1) [13,14]:

(1) $$\mathrm{NDVI}=\;\frac{R_{790}-R_{660}}{R_{790}+R_{660}}$$

where R_790_ = digital number (DN) (0–65535) of the NIR channel (770–810 nm); R_660_ = DN (0–65535) of the red channel (640–680 nm).

### 2.5. Plant Sampling and Chlorophyll Analysis

Eleven spots in the field were randomly chosen to collect samples. Thirty buckwheat plants were collected from each spot within the field after the images were taken. Each bundle of plant samples from each collection site were wrapped in the plastic bags separately and stored in an ice box.

Chlorophyll extraction and measurements were performed according to the method of Chappelle et al. [15]. One hundred milligrams of the sample was immersed in 10 mL of dimethyl sulfoxide (DMSO), and the pigment was extracted for 48 h under dark conditions at 30 °C. The absorbance was measured at 664, 648 and 470 nm using a spectrophotometer (UV-1800, Shimadzu Corporation, Tokyo, Japan). The extracted chlorophyll content was calculated using the following equations (Equations (2)–(4)):

Chlorophyll a = 12.25A664 nm − 2.79A648 nm(2)

Chlorophyll b = 21.50A648 nm − 5.10A664 nm(3)

Total chlorophyll = Chlorophyll a + Chlorophyll b(4)

where A is absorbance and pigment concentration calculated as μg·mL^−1^ of extract.

### 2.6. Statistical Analysis

Simple linear regression analysis was done to verify the linear relationship between the total chlorophyll and the NDVI. The simple linear regression model is described mathematically by following Equation (5):

*y_i_* = *β*_0_ + *β*_1_ × *x_i_* + ε*_i_* (5)

where *y_i_* is the response variable of the model obtained by the average of the two replicates of total chlorophyll per sample, *x_i_* is the explanatory variable that represents the i-th observed value of one of the indices calculated in each sample, *β_0_* is the intercept of the model, *β_1_* is the slope of the straight line, and ε*_i_* is the random error of the model which is assumed to follow Gaussian distribution with mean equal to zero and variance equal to σ^2^.

Parameters of the simple linear regression model were estimated by the least square method [16]. Ninety-five percent confidence intervals for conditional mean of the total chlorophyll given the level of one of index E(*y_i_*|*x_i_*) were drawn to know the precision and the response mean was estimated [16]. The limit of confident intervals is given by Equation (6):

(6) $$y={\hat{y}}_{i}\pm t_{n-2}\sqrt{\hat{\sigma^{2}}\left(\frac{1}{n}+\frac{{\left(x_{i}-\bar{x}\right)}^{2}}{\left(n-1\right)s_{x}^{2}}\right)}$$

where ${\hat{y}}_{i}$ is the predicted value, $t_{n-2}$ is the 97.5 quantile of t student with n-2 degree of freedom, $\hat{\sigma^{2}}$ is the mean square of the error, and $s_{x}^{2}$ is the sample variance of explanatory variable.

F statistics from analysis of variance were computed to test the null hypothesis *H*_0_: *β*_1_ = 0 and values of coefficient of determination were used as a goodness-of-fit measure.

Additionally, due to the fact there are more than one replicate of total chlorophyll per sample, a lack-of-fit test was performed to verify the assumption that the straight line is the correct model to describe the relationship between total chlorophyll and the indexes. In that case, the statistical model is given by Equation (7):

y_ij_ = β_0_ + β_1_ × x_ij_ + ε_ij_ (7)

where *y_ij_* represents the total chlorophyll of i-th sample and j-th replicate and *x_ij_* represents the observed value of one of the indexes measured at i-th sample and j-th replicate.

The F statistics of the lack-of-fit test is defined by Equation (8):

(8) $$F=\;\frac{Mean\;Square\;Lack\;of\;Fit}{Mean\;Square\;Pure\;Error}$$

## 3. Results

**Figure 5:**
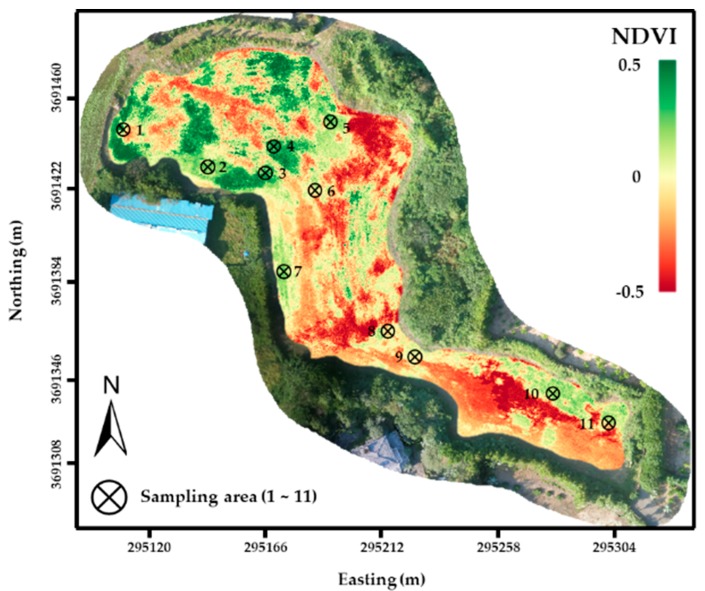
Generated normalized difference vegetation index (NDVI) maps in test site.

Field maps with RGB and NDVI data were generated (Figure 5). Vegetative area shown as the green color in the RGB map was well matched with the NDVI considering the typical value of the NDVI ranges from 0.1 to 0.6 [17]. Since this field was severely damaged by typhoon KONG-REY, on 7 October 2018, bare soils were exposed and are expressed as the red color.

**Figure 6:**
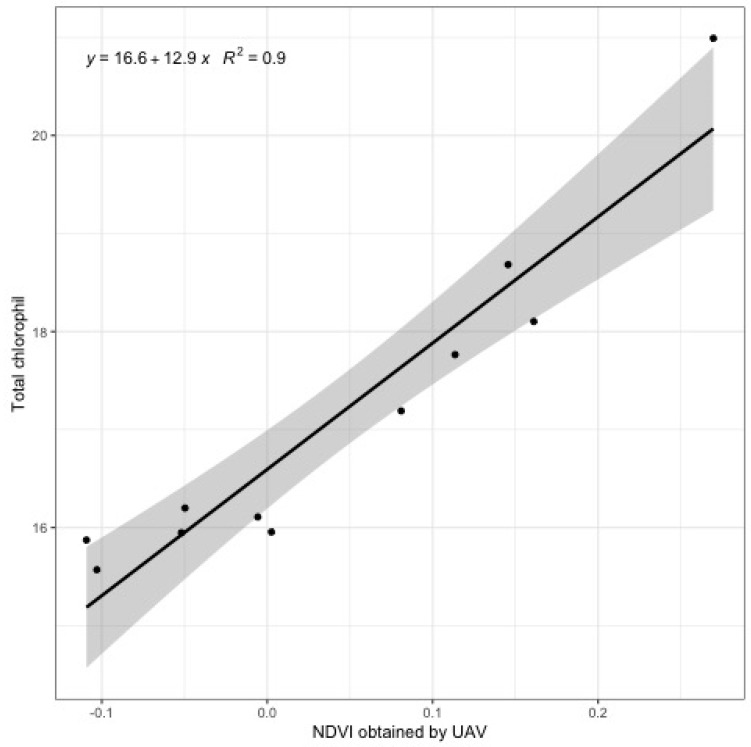
Simple linear regression analysis for comparison of the normalized difference vegetation index (NDVI) value determined by the unmanned aerial vehicle (UAV)-based image and ground truth chlorophyll content (μg·mL^−1^) from 11 sample sites. Shadow indicates 95% confident interval.

Simple linear regression analysis to investigate the relationship between total chlorophyll and NDVI shows that there is statistical evidence to support the statement that the relationship between total chlorophyll and the NDVI are linear (Table 2). Additionally, results from the F lack-of-fit test indicates the assumption that the straight line is a reasonable model is correct (Table 3). High values for coefficient of determination (0.90) and narrow confident intervals for mean response was found for models where the explanatory variable is represented by the NDVI. It means the high goodness-of-fit model and that the response mean conditional to the levels of explanatory variable was estimated with high precision although only small numbers (two replications of chlorophyll data with 11 sites) were used. Indeed, the chlorophyll values fit well within the 95% confidence interval (Figure 6). These results mean that the NDVI obtained from a low-resolution camera with a small sample size could be applied to estimate the chlorophyll content of the target crop in the field.

## 4. Discussion

The purpose of this study was to investigate if the images taken from a low-resolution camera mounted on a low-cost UAV could estimate chlorophyll content in the crop planted in an open field condition. If it is so, this method would be used as the precision agriculture and an acceptable manner could be achieved. Based on the results, the current study demonstrates that the chlorophyll content of buckwheat can be estimated using the NDVI providing simple but clear results. The methods in the current study are relatively easy and would be very useful in terms of accuracy, time saving, and cost-effectiveness for measuring chlorophyll contents for fertilizing N at the proper timing, which could be applied to other crops such as corn, soybeans. The most beneficial application would be applying N based on the NDVI which reflects the chlorophyll content in the crop in a large and open field. This method could even be applied to phenotype N uptake for pre-selection in the breeding program as well as to diagnose plant health with other indices derived from the NDVI such as GNDVI (Green Normalized Difference Vegetation Index) [18], ENDVI (Enhanced Normalized Difference Vegetation Index) [19], SAVI (Soil Adjusted Vegetation Index) [20], and OSAVI (Optimized Soil Adjusted Vegetation Index) [21]. However, difference in leaf structure may necessitate species-specific calibration equations [22]. Thus, it may be worth investigating other indexes derived from the NDVI with the same data set for fine-tuning the model in the future.

Undoubtedly, it would be good to be fully equipped with high-resolution camera and cutting edge UAVs operated with a highly skilled workforce to have better results for more precise agriculture. However, the results in the current study demonstrated that our method is enough to estimate chlorophyll content in the scale of the field. This could be helpful to apply N fertilizer application where the chlorophyll value expressed by NDVI is low. In this sense, this study has its own unique value for those who want to utilize UAV for their research program with tight budgets. Furthermore, even small changes for improving NUE could be helpful to sustainable agriculture by protecting fragile environments such as Jeju Island.

## Figures and Tables

**Table 1:** Details of UAV flight in this study.

Flight Date	Flight Speed (m·s^−1^)	Flight Altitude (m)	The Number of Images	Ground Sample Distance (GSD) (cm·Pixel^−1^)	Flight Time	Illumination	Wind (m·s^−1^)
13 October 2018	2	80	2135	8.12	4–5 pm	Clear	1.5

**Table 2:** Analysis of variance table computed for each simple linear regression model fitted to data of total chlorophyll and infrared vegetation index: normalized difference vegetation index (NDVI).

Source of Variation	df	Sum of Square	Mean Square	F Statistics	*p* Value
Regression	1	24.6246	24.6246	80.631	<0.0001
Residuals	9	2.7486	0.3054		

**Table 3:** Analysis of variance table from lack-of-fit test computed for each simple linear regression model fitted to data of total chlorophyll and infrared vegetation index: normalized difference vegetation index (NDVI).

Source of Variation	df	Sum of Square	Mean Square	F Statistics	*p* Value
Lack of Fit	9	5.4972	0.6108	0.1808	0.992
Error Pure	11	37.168	3.3789		
